# British anti-Lewisite (BAL) reduces the severity of systemic and local responses of the eye after exposure to the chemical warfare agent surrogate for Lewisite, phenylarsine oxide (PAO)

**DOI:** 10.1016/j.toxrep.2025.102153

**Published:** 2025-10-28

**Authors:** Sarbani Hazra, Aditya Konar, Robb Welty, Uday B. Kompella

**Affiliations:** a Department of Veterinary Surgery & Radiology, West Bengal University of Animal & Fishery Sciences, Khudiram Bose Sarani, Kolkata, India; b CSIR-IICB, Jadavpur, Kolkata, India; c Department of Pharmaceutical Sciences, University of Colorado Anschutz Medical Campus, Aurora, CO 80045, USA; d Department of Biochemistry and Molecular Genetics, University of Colorado School of Medicine, Aurora, CO 80045, USA; e Department of Ophthalmology, University of Colorado Anschutz Medical Campus, Aurora, CO 80045, USA; f Department of Bioengineering, University of Colorado Anschutz Medical Campus, Aurora, CO 80045, USA; g Colorado Center for Nanomedicine and Nanosafety, University of Colorado Anschutz Medical Campus, Aurora, CO 80045, USA

**Keywords:** Arsenical vesicants, British anti-Lewisite (BAL), phenylarsine oxide (PAO), chemical warfare agents (CWAs), Cornea, and Retina

## Abstract

Arsenical ocular toxicity is acute, painful, and aggressive. Although British anti-Lewisite (BAL) is an approved therapy for systemic arsenical toxicity, remedial measure for ocular exposure of arsenicals is still an unmet need. This study evaluated the efficacy of BAL as topical drop for ameliorating the pathogenesis incited by phenylarsine oxide (PAO, surrogate for lewisite), a chemical warfare agent. The binding of BAL to arsenic and calcium and zinc, two essential cellular minerals was determined using Isothermal Titration Calorimetry (ITC). Ex vivo, mouse corneas were tested with various concentrations of BAL (0.1 %, 1 %, and 5 %). Injury was induced ex vivo using PAO, 25 µg/5 µL, and rescue was evaluated with 1 % BAL. In-vivo, injury to mouse cornea was induced with PAO 100 µg/5 µL and rescue was evaluated by 1 % BAL. All eyes were assessed for physical symptoms by examination under slit lamp biomicroscope, anterior segment optical coherence tomography (AS-OCT), corneal thickness, and histopathological changes. BAL bonded strongly with arsenic but negligibly with calcium and zinc. Ex-vivo cornea, response with 1 % BAL was graded superior to higher concentration. One of eight mice with PAO injury survived versus all survivals in the PAO+BAL group after injury. Topical use of BAL mitigated the exacerbated ocular response exhibited by PAO injury. Histology revealed better preservation of retinal architecture in BAL treated mice. 1 % BAL alleviates PAO induced fatality in mice. The rescue in the posterior segment pathogenesis was remarkable. BAL is a promising decontaminant for ocular arsenical exposure and warrants further investigation.

## Highlights

• Evaluated the efficacy of BAL as an eye drop in a murine model for ameliorating the pathogenesis incited by phenyl arsine oxide (PAO).
• Assessments included histopathology as well as eye examinations under slit lamp biomicroscope and anterior segment optical coherence tomography (AS-OCT).
• The results indicated that BAL is a promising decontaminant for ocular surface injury caused by arsenical exposure.
• Histology revealed better preservation of retinal architecture in BAL treated mice.
• PAO induced fatality in 87.5 % of mice in 48 h. With 1 % BAL eye drop, all mice survived 48 h.

## Graphical Abstract

**Figure:**
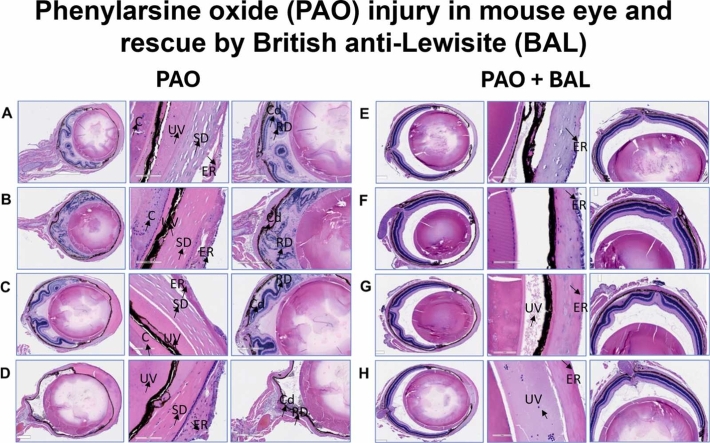

## 1 Introduction

Vesicant exposure is a major concern in respect to corneal injury, specifically lewisite (LEW), an organic arsenical, dichloro (2-chlorovinyl)-arsine, which exhibits fast penetrability through the cornea, injury is acute, remarkably inflammatory and has vision threatening consequences [1], [2], [3]. Literature also reports deaths due to lewisite exposure on the skin which highlights the fatality caused by the agent and the need to investigate further the local and systemic manifestations of ocular exposure [4], [5]. Several agencies National Institute of Health (NIH), Public Health Emergency Medical Countermeasures Enterprise (PHEMCE), Department of Defense, Biomedical Advanced Research and Development Authority (BARDA) have enlisted lewisite with top priority for studies on its countermeasures as a strategy for national preparedness. To avoid long drug development strategies these agencies have also advocated the repurposing of FDA approved drugs as relevant medical countermeasures or antidotes for vesicant injuries [6], [7]. Although emphasis is being directed towards understanding pathogenesis and identifying suitable therapy for lewisite ocular injury [8], to date there has been no drug approved as a countermeasure for this agent in the eye. British anti-Lewisite (BAL) is the FDA approved drug for systemic lewisite exposure [9]. We strongly believe that the topical instillation of BAL on the corneal surface immediately after exposure to arsenic based chemical warfare can potentially chelate out the agent, limit the toxic effect and aid the mainstay treatment regimen where the intervention time is critical. Additionally, lipophilic BAL can penetrate compartments where toxic metal ions are concentrated.

A recent study has demonstrated the safety and efficacy of BAL in chelating cobalt released from hip prosthesis Chinedu C. Ude et al. [10]. But available knowledge for ocular use is very limited. Hugh’s paper from 1947 reported the effective topical use of BAL, for less severe corneal lewisite exposure at various concentrations and in several species (e.g., rabbit, monkey, and humans) [8], [10]. It also mentions the side effects of BAL on the cornea, which includes the formation of a drug sheet that compromises with the corneal clarity in rabbits. Exposure in humans has been reported to cause a stinging sensation, blepharospasm, epiphora, and conjunctival congestion. Though the exact mechanism of BAL induced cellular dysfunction has been poorly documented, interference with Ca^2 +^ transport has been reported in the brain tissue [11]. Surprisingly, there has been no organized study thereafter to improvise the use of BAL as a suitable decontaminant for arsenical injury on the ocular surface or to steer it towards a single or a combinational therapy.

The previous studies have focused on the treatment of post vesicant injury to the eye with therapeutics (e.g., Goswami et al., 2016) [12], this study aims at investigating the decontamination activity of BAL as a chelator of arsenic, and its efficacy in ameliorating the injury inflicted by phenylarsine oxide (PAO), a less toxic surrogate of lewisite (LEW) [12]. PAO has demonstrated comparable cytotoxicity, corneal injury, and like lewisite its exposure to the skin has incited erythema, edema, and micro blisters, with systemic affection of the lungs [3], [13], [14]. The rationale of using BAL is to neutralize arsenic, which may result in arresting the injury to the sensitive structures of the eye and dampen the pathogenesis. This is the first study to investigate the efficacy of BAL in mitigating severe ocular injury incited by PAO, a surrogate of lewisite.

## 2 Materials

### 2.1 Chemicals

The Lewisite surrogate, Phenylarsine oxide (PAO), and British anti-Lewisite (BAL) (also known as dimercaprol or 2,3-dimercaptopropan-1-ol) were obtained from Merck, Rahway, NJ, USA. Calcium chloride (CaCl_2_), Zinc Chloride (ZnCl_2_), Sodium meta arsenite (NaAsO_2_), and sodium fluorescein (NaF) was purchased from Sigma-Aldrich, St. Louis, MO, USA. Potassium chloride, potassium phosphate dibasic, sodium chloride, and sodium phosphate dibasic were purchased from Fisher Scientific, Pittsburgh, PA, USA. Ethanol was purchased from Decon Labs, Inc., King of Prussia, PA.

### 2.2 Animal model

Ex vivo studies were conducted using eyes from C57BL/6 mice, which were received immediately after being terminated during a routine Euthanization training program for all individuals intending to use animal for research, conducted by the Institutional Animal Care and Use Committee (IACUC) in conjunction with the Office of Laboratory Animal Resources (OLAR) at the University of Colorado Anschutz Medical campus, and which was in compliance with all policies for animal usage. Only mouse eyes without gross external anomalies were included in the study.

In-vivo studies were conducted using C57BL/6 mice obtained from Charles River Biosciences, Wilmington, MA. All studies were conducted in accordance with ARVO guidelines for Vision and Ophthalmic research and approved by the Institutional Animal Care and Use Committee of the University of Colorado Anschutz Medical campus, at Aurora, CO using the IACUC-Protocol number-01125.

## 3 Methods

***As described in the methods below, this study assessed whether BAL binds to arsenic but not calcium and zinc and/or whether BAL mitigates ocular injury caused by PAO in the ex-vivo and in-vivo settings.***

### 3.1 Molecular interaction by Isothermal Titration Calorimetry (ITC) experiments

ITC experiments were performed with an ITC200 (Malvern Panalytical, Westborough, MA) and consisted of an initial 0.4 µl injection (data point was removed before analysis), followed by 18 sequential 2 µl injections. To accommodate slower reaction kinetics, the inter-injection wait time was extended from 180 to 300 s. All experiments were performed in a buffer background of 100 mM, pH 7.5, HEPES at 25°C. In all experiments BAL containing solutions (0.25 mM) were placed in the measurement cell of the ITC, and the ionic solutions of ZnCl_2_, CaCl_2_, and sodium meta-arsenate (NaAsO_2_) (all at 2.5 mM) were loaded into the syringe.

ITC thermograms were pre-processed with NITPIC [15], [16], for improved quantification of titration injection peaks. Processed data was globally fit to a 1:1 binding isotherm with SEDPHAT [17], the fits held all parameters in common between data sets, with the notable exception of “active fraction”. The analyzed data was then plotted using the GUSSI software suite [18].

### 3.2 Ex vivo evaluations and treatments for corneal responses

#### 3.2.1 Corneal responses to different concentrations of 0.1 %,1 %, and 5 % BAL in ex vivo eyes

The corneas of the euthanized mice (N = 6, in each group) were treated with 5 µL of either 0.1 %, 1 %, 5 % BAL or phosphate buffered saline solution (PBS, pH 7.4). The response of the corneal surface to various concentrations of BAL and PBS was examined by the methods described below.

***Examination under Slit Lamp Biomicroscope***

Thirty minutes after the instillation of BAL, fluorescein dye strips were used to instill the dye onto the corneal surfaces and then washed out with 2 mL of sterile saline solution at room temperature. The eyes were examined using slit lamp bio-microscope (Topcon, Japan) under bright and cobalt blue light settings.

***Evaluation of eyes with Anterior Segment OCT (AS-OCT)***

The eyes were hereafter evaluated with anterior segment spectral domain OCT (Bioptigen Envisu R2200-HR SD-OCT) to determine the corneal thickness and corneal section images were also captured in all the eyes. After capturing images, the OCT instruments caliper was placed in the thickest region of the corneal section, and the thickness was recorded.

#### 3.2.2 Phenylarsine oxide induced injury and 1 % BAL antidote in the ex vivo mouse eyes

The cornea of four eyes from euthanized mice were instilled with 5 µL of PAO at a concentration of 25 µg/5 µL 100 % ethanol. The eyes were rinsed after 30 min, and a fluorescein dye test was conducted. The eyes were examined under a slit lamp biomicroscope, and corneal thickness was evaluated with AS-OCT.

In another batch of four eyes, the corneas were instilled 5 µL with PAO at a concentration of 25 µg/5 µL 100 % ethanol and after 5 min, 1 % BAL (5 µL) was instilled on all the four eyes. Thirty minutes after PAO injury, the eyes were subjected to a fluorescein retention test, and eye examination was conducted under slit lamp biomicroscope and the measurement of corneal thickness was done by AS-OCT, as per the methods described above.

### 3.3 In-vivo evaluations of corneal responses

#### 3.3.1 In-vivo corneal responses for PAO injury and 1 % BAL antidote

Both eyes of the mice were examined prior to the procedures to rule out pre-existing abnormalities. The mice received a single dose of carprofen at 5 mg/kg/SC, 30 min prior to the procedures.

#### 3.3.2 In-vivo corneal induced injury with PAO

The mice (N = 8, 4 male and 4 female) were anaesthetized with 3 % isoflurane and were maintained with 1 % isoflurane when required. While under anesthesia, left eyes were treated with PAO 100 µg/5 µL 100 % ethanol and right eyes received only 5 µL of 100 % ethanol. After inducing the injury, each mouse was immediately placed into its cage and monitored for signs of ocular irritation upon recovery. Irritation was evaluated by observing the animal’s tendency to wipe their eyes. It was evaluated by counting the number of times the eye was wiped with ipsilateral paw in 30 s [19]. Thirty minutes after the PAO was instilled on the ocular surface, the mice were anesthetized using 3 % isoflurane, also 5 µL of fluorescein dye was instilled on the corneas of both eyes, and then they were washed with 2 mL of sterile saline. At this point, eyes were evaluated under a slit lamp biomicroscope, for fluorescein retention, eyelid edema, scoring it from 0 to 3 [20], corneal haze, from 0 to 4 [21], and this was followed by evaluation with OCT to assess corneal thickness (mm). These examinations were repeated under 3 % isoflurane anesthesia after 48-hr and 7-days post injury.

#### 3.3.3 In-vivo corneal injury induced with PAO followed by topical application of 1 % BAL antidote

The mice (N = 8, 4 male and 4 female) were anaesthetized with 3 % isoflurane and maintained with 1 % isoflurane when required. While under anesthesia, left mice eyes were treated with PAO 100 µg/5 µL 100 % ethanol and right mice eyes received only 5 µL of 100 % ethanol. Five minutes after the PAO injury, each eye received 5 µL of 1 % BAL antidote. Following recovery from anesthesia, eye wiping was evaluated by counting the number of times the eye was wiped with the ipsilateral paw in 30 s. Thirty minutes after the initial PAO instillation onto the ocular surface, the mice were again anesthetized with 3 % isoflurane and additional examinations were performed in a comparable manner as those described in the previous section. These examinations were repeated under 3 % isoflurane anesthesia at 48-hr and 7-days post PAO injury and the application of the 1 % BAL antidote. Subsequently, the mice were euthanized by CO2 asphyxiation at various time points (e.g., 48-hr and 7-days). At the end of the experiments, whole eyes were preserved in Davidson’s fixative solution for one of histology’s principal tissue staining techniques using a Hematoxylin-Eosin (HE) stain. This procedure was performed by an outside histological processing company, (Excalibur Pathology, Inc., in Norman, OK). Briefly, upon receipt of the eyes by Excalibur, each specimen was given a unique lab accession number. A central horizontal section from eyes of species with macula containing pupil, optic disc, and macula is taken unless other pathology is noted. Those species like mice with no macula are marked at the superior limbus to show superior/inferior PO sections. At this point, the central section or whole eye is placed in a cassette that has been labeled with its unique lab identifier stamped on it. The cassette is then sent through the paraffin process in which the specimen is embedded in the paraffin inside the cassette. Next, sections are taken at 4–6 µm depending on the study species. Slides are first air dried and then moved to an oven for additional drying. Once they are dry the slides are deparaffinized, rehydrated, and stained with hematoxylin and eosin staining.

By using this histological processing, the hematoxylin stains the cell nuclei a purplish-blue color, while the eosin stains the extracellular matrix (collagen) and cytoplasm pink, during the process other cell structures take on different shades and hues or different combinations of these colors. Red blood cells are stained with an intense red color. In this way, a pathologist can differentiate between cell parts, and the color pattern provides a general layout, distribution, and overview of tissue sample’s structures. This pattern recognition is either observed by an experienced researcher or by using software (e.g., Aperio ImageScope, revision Q from 2017, Leica Biosystems, Nussloch, Germany) or both for histologic information.

### 3.4 Statistical analysis

Two groups were compared using the student’s *t*-test. Multiple groups were compared using one-way ANOVA for continuous data (corneal thickness), which was determined to be normally distributed based on Shapiro-Wilk test and using Kruskal-Wallis test for discrete (eye wipe counts) or categorical data (lid edema and corneal haze). Differences were considered statistically significant at p < 0.05.

## 4 Results

### 4.1 Interaction between BAL and Arsenite from ITC experiments

**Fig. 1:**
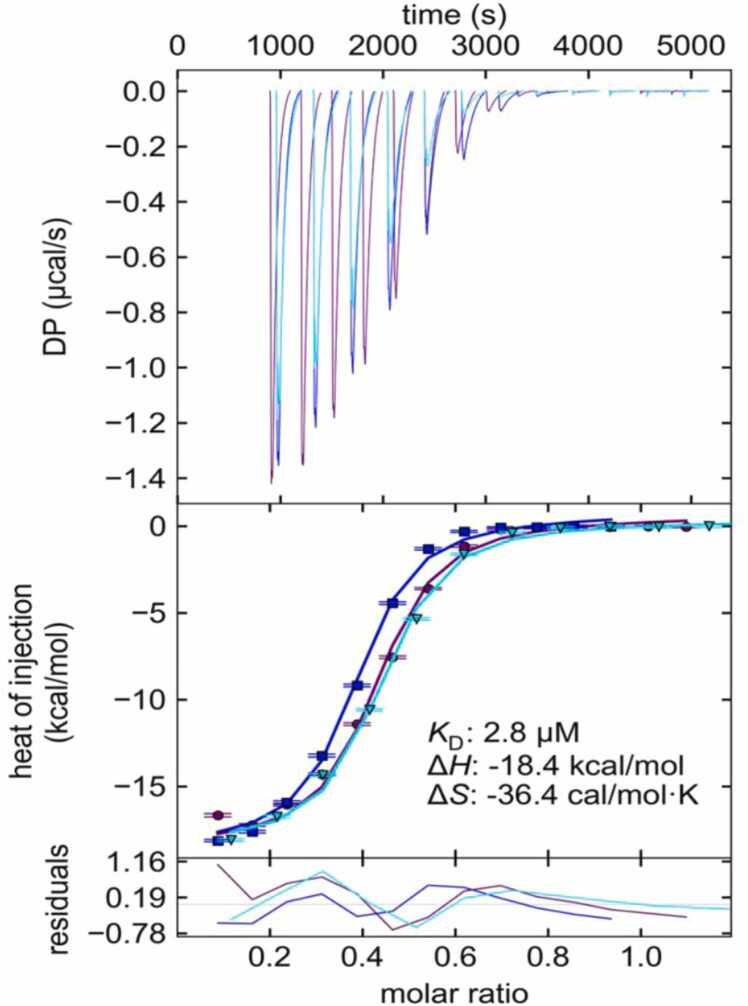
Isothermal Titration Calorimetry (ITC) of arsenite reacting with BAL in triplicate. Top) Thermograms of exothermic peaks resulting from arsenite titration into BAL solution. Middle) Isotherm plot, showing the heats of interaction, global fit curves, and thermodynamic values Kd, dH, and dS generated from the global fit. Bottom) residual curves showing the difference between data and fit curves.

The ITC experiments show that BAL interacts with arsenite with an affinity of 2.8 micromolar. Further, the change in enthalpy and entropy between unbound and bound states are −18.4 kCal/mol, and −36.4 cal/MolK, respectively. From the data in (Fig. 1), we can observe two interesting phenomena. The first is that the isotherm curves do not completely overlap, and the second is that the reaction reaches an apparent saturation with a molar stoichiometry nearing 0.5 BAL/Arsenite. Originally, we surmised that a single BAL was binding two arsenite molecules; however, there is no sensible chemical mechanism for that reaction.

We believe that the most likely explanation is that the BAL molecules in the solution exist in multiple redox states. This is not uncommon and usually expected for compounds with thiol groups in close proximity [22]. For this reason, when the isotherms were globally fit, and we let an “active fraction” parameter float, reasoning that oxidized BAL molecules should be incapable of interaction, rendering them a part of the “inactive fraction”. This observation and rationale do not significantly impact on our other results, as BAL is in significant excess.

**Fig. 2:**
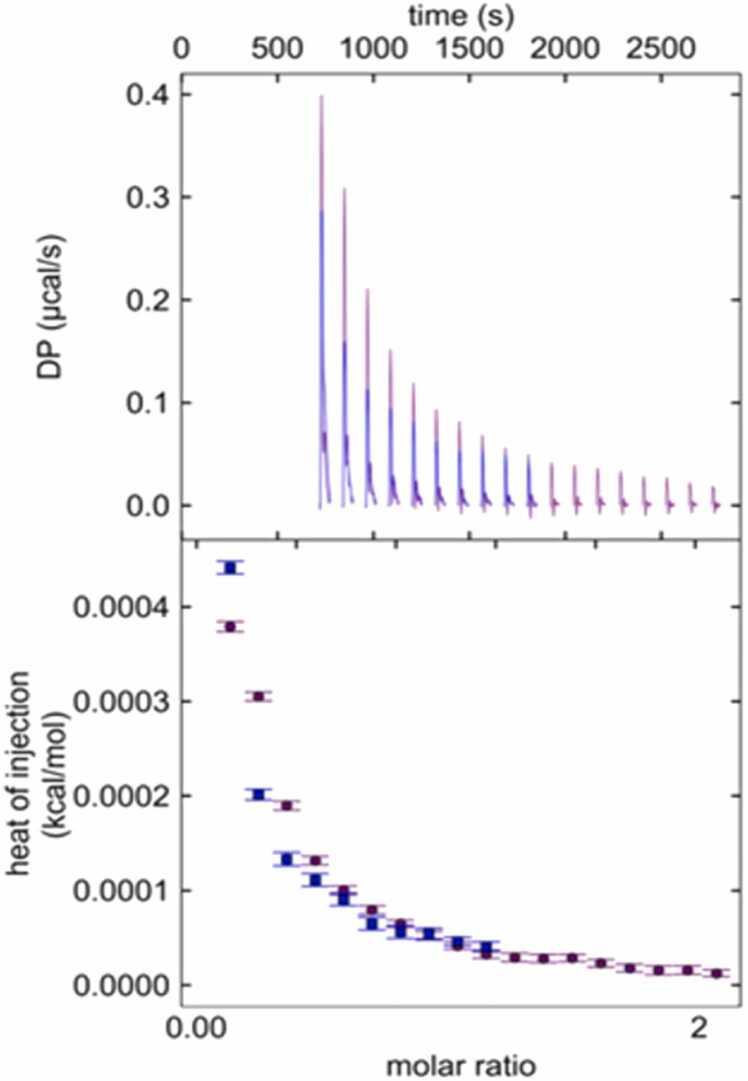
Isothermal Titration Calorimetry (ITC) of zinc chloride being titrated into BAL (purple) and experimental buffer (blue). Top) Thermograms of endothermic peaks showing the heat of dilution of zinc chloride. Bottom) Isotherm plot, showing the heats of dilution of zinc chloride being the same + /- BAL. Curves were not fit, as standard binding isotherms only are applicable for binding interactions. The X axis represents the molar ratio of Zinc chloride to BAL, as there is no applicable molar ratio for the buffer condition.

The other ionic solutions, zinc and calcium, indicate no interaction with BAL. There was no appreciable heat change with calcium chloride. Injections of zinc chloride did produce endothermic peaks, but they were indistinguishable between injections into BAL or injections into buffer (a negative control), (Fig. 2). This suggests that the identity of the cation is important and only affects ions that can undergo appropriate REDOX chemistry.

### 4.2 Ex vivo corneal responses

#### 4.2.1 Ex vivo corneal responses of mice eyes to different concentrations, 0.1 %, 1 % and 5 % of BAL in solution

Gross examination of the eyes under the slit lamp biomicroscope revealed a drug sheet on the corneal surface in eyes treated with the various concentrations of BAL, thereby affecting the corneal clarity, which was further reduced with higher application concentrations, these effects were not observed with PBS treated eyes. One out of six (16 %) of the eyes in both the PBS and 0.1 % BAL treated groups showed fluorescein retention. On the other hand, two out of six (33 %) of the corneas in 1 % BAL treated group had fluorescein retention and finally four out of six (66 %) of the corneas showed fluorescein retention in 5 % BAL treated group (Supplemental Figure S1, Panels 1 – 4).

**Fig. 3:**
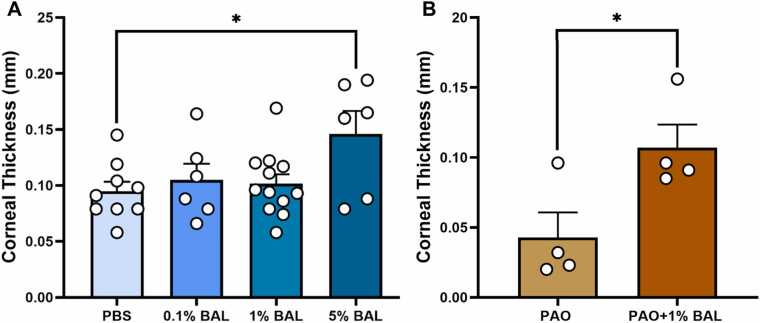
A and 3B. Bar graph representing the changes in corneal thickness of ex-vivo mice cornea treated with different concentrations of BAL, and changes inflicted by PAO injury and its arrest using PAO+ 1 % BAL. 3 A) Graph shows the corneal thickness (mm) measured by OCT, with 0.1 % (n = 6) and 1 % BAL (n = 12) versus PBS (n = 9), with the result not significant at p > 0.05. Additionally, corneal thickness (mm) measured by OCT shows an apparent increase in thickness with 5 % BAL (n = 6) versus PBS (p < 0.05). 3B) The bars show that the corneal integrity was preserved in eyes that received PAO+ 1 % BAL versus the thinning of cornea in the PAO only treated corneas. The data represents mean ± s.e.m. *The result is statistically different at p < 0.05, (n = 4).

There was no significant difference (p > 0.05) in the corneal thickness as determined by OCT between PBS, 0.1 %, and 1 % BAL treated corneas. Corneal thickness with 5 % BAL was significantly higher versus PBS (p < 0.05), indicating 5 % BAL was influencing the corneal surface, (Fig. 3).

#### 4.2.2 Ex vivo mice cornea responses show that 1 % BAL offers protection to PAO induced injury

Gross examination under the bright light and cobalt blue light settings of the slit lamp biomicroscope showed positive fluorescein retention in four out of four (100 %) of the eyes injured with 5 µL PAO. The AS-OCT images suggested thinning of the corneas in PAO treated eyes. Histological eye tissue sections showed loss of corneal epithelium in regions of fluorescein retention in the clinical images. The concomitant use of PAO and BAL shows the apparent lack of fluorescein in one out of four corneas. Very mild fluorescein retention has been noted in two out of four corneas and apparent dye retention and peeling of epithelium in the fourth cornea. The loss of corneal epithelium in histopathological sections corroborates and matches the clinical observations, (Supplemental Figure S2, Panels 1 and 2). Corneal thickness in the BAL treated eyes were preserved versus PAO injured corneas. There was a significant difference in corneal thickness in the corneas, which received BAL following PAO injury versus only PAO injury, (Fig. 3).

### 4.3 PAO corneal injury leads to mortality in mice by 48-hr with aggressive anterior / posterior segment responses in eyes

**Fig. 4:**
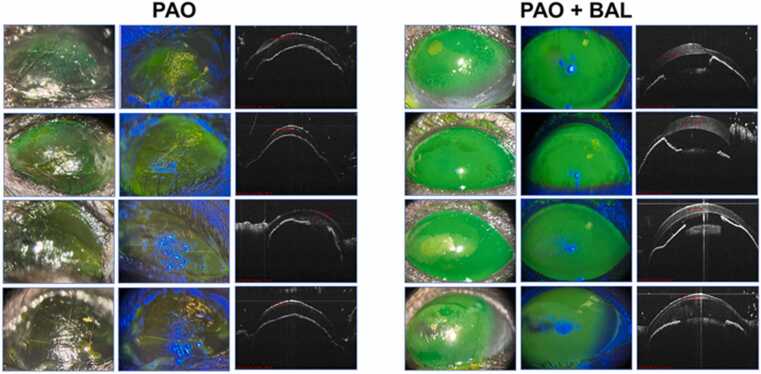
Representative Slit lamp and OCT images of in-vivo mice eyes injured with PAO and treated with BAL. Slit Lamp and OCT images of in-vivo mouse eye injury with 100 µg PAO and its countermeasure using 1 % BAL. The clinical evaluations by slit lamp biomicroscope and OCT at the 48-hr time point are shown in 100 µg PAO treated eyes. The corneas were positive for fluorescein retention and appeared severely dry and lusterless without normal contour, indicating a deeper stromal involvement. The 100 µg PAO + 1 % BAL treated eyes show extensive fluorescein retention over the cornea, while the cornea retained a normal contour and luster, indicating a superficial corneal involvement.

**Fig. 5:**
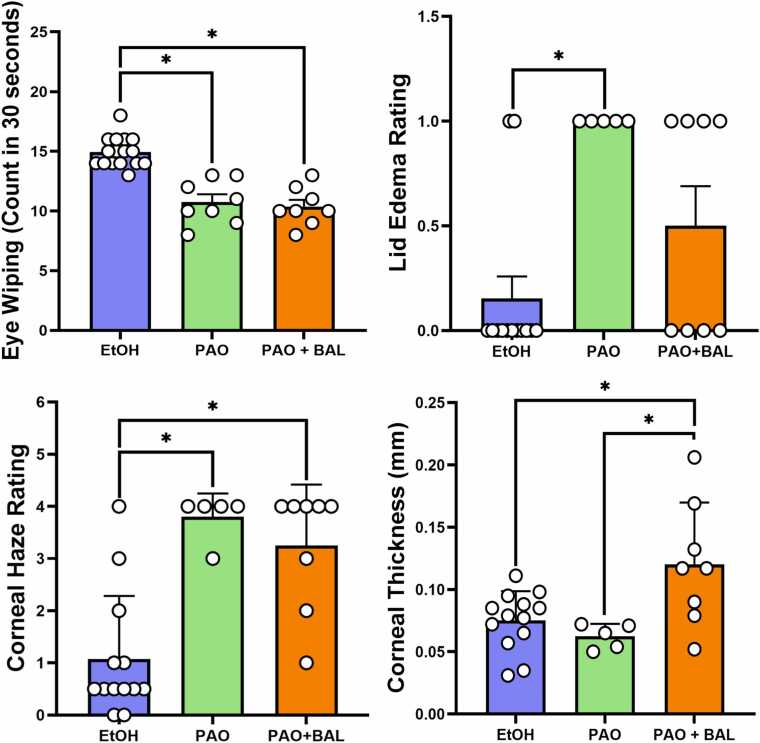
Bar graphs represent the effects of 100 µg PAO, 100 µg PAO+ 1 % BAL, or 100 % ethanol on eye wiping frequency, lid edema, corneal haze, and corneal thickness. All parameters were measured 48-hr after injury, except eye wiping frequency, which was measured soon after treatments. The data represents mean ± s.e.m. *The result is statistically different at p < 0.05.

**Fig. 6:**
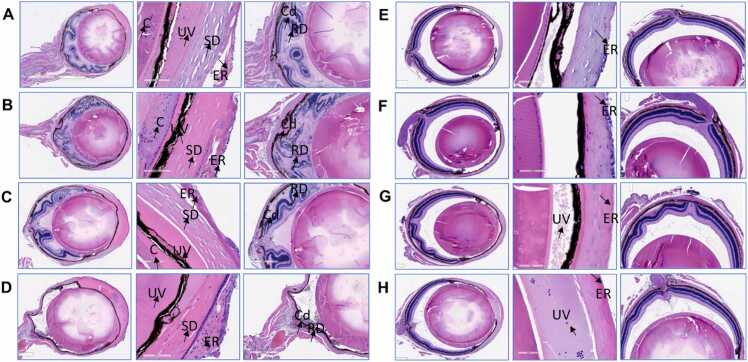
**Images A to H.** The histological sections of in-vivo study with mice eyes, showing the whole eye (Left), anterior segment (Center), and posterior segment (Right) of PAO injured (A-D) and BAL treated eyes(E-H) at 48-hr post injury. Eyes treated with either 100 µg PAO or a combination of 100 µg PAO + 1 % BAL show a very distinctive ocular tissue response. The 100 µg PAO treated eyes showed severe anterior and posterior segment injury, while there was only an anterior segment change in 100 µg PAO + 1 % BAL treated eyes. Additionally, the posterior segment shows an obvious rescue from degeneration and detachment. A.) Left – Whole eye Animal 1 - male, expired 48-hr post injury. Center-anterior uveitis, cataract, stromal degeneration, and the erosion of epithelial layer. Right – Choroiditis, retinal degeneration, detachment, and posterior uveitis. B.) Left – Whole eye Animal 2 – male, expired 48-hr post injury, Center - anterior uveitis, cataract, stromal degeneration, erosion of epithelial layer. Right – choroiditis, severe retinal degeneration, detachment, and posterior uveitis. C.) Left - Whole eye Animal 4 – male, expired 48-hr post injury. Center - anterior uveitis, cataract, stromal degeneration, erosion of epithelial layer. Right - choroiditis, retinal degeneration, detachment, and posterior uveitis. D.) Left – Whole eye Animal 5 – female, expired 48-hr post injury. Center- anterior uveitis, cataract, stromal degeneration, and erosion of epithelial layer. Right - choroiditis, severe retinal degeneration, retinal detachment, and posterior uveitis. E.) Left – Whole eye Animal 9, male, euthanized at 48-hr. Center - loss of superficial epithelium, mild stromal dystrophy, mild reaction in anterior chamber, and the lens were normal. Right - normal choroid, retina normal and attached, and vitreous normal appearance. F.) Left – Whole eye Animal 10 - male, euthanized at 48-hr. Center - mild erosion of superficial epithelium, no stromal dystrophy, no reaction in anterior chamber, and the lens was normal. Right - normal choroid, retina normal and attached, and vitreous normal appearance. G.) Left – Whole eye Animal 13 - Female, euthanized at 48-hr. Center - epithelial loss evident, moderate anterior chamber reaction, and cataractous lens. Right - normal choroid, retina normal, and attached vitreous with normal appearance. H.) Left - Whole eye Animal 14 - Female, euthanized at 48-hr. Center - epithelial loss evident, high anterior chamber reaction, and normal lens. Right - normal choroid, retina normal, and attached vitreous with normal appearance. Marked by arrow and abbreviations, Uveitis (UV), Stromal degeneration (SD), Cataract (C), Epithelial erosion (ER), Choroiditis (Cd), Retinal Degeneration and detachment (RD).

Three mice died within 24-hr, and four mice died within 48-hr after the topical treatment of cornea with 100 µg of PAO. Surprisingly, one mouse survived for 7 days. The clinical and histological features demonstrate aggressive anterior and posterior segment response in all eyes (Table 1). PAO injured eyes exhibited significantly higher eye lid edema and corneal haze versus ethanol treated eyes at 48-hr time point (Fig. 5). There was no significant difference in lid edema between eyes treated with PAO+BAL versus vehicle control group. Corneal thickness for PAO+BAL was significantly higher versus ethanol and PAO (Fig. 4, Fig. 5, Fig. 6). Further, eye wiping frequency was the highest (p < 0.05) in ethanol treated eyes (Fig. 5).

**Table 1:** Effect of PAO with or without BAL on eye injury and animal survival.

**Description** **of Injury**	**Number** **of mice**	**Post Treatment (Morbidity/Mortality)**	**Histopathological Observations from Enucleated Eyes**	
			**Anterior Segment**	**Posterior Segment**
**1.)** Ipsilateral eye received PAO 100 µg / 5 µL ethanol. Contralateral eye received 5 µL of 100 % ethanol.	8*	Acute mortality was observed after PAO topical drops. 7 mice injured with PAO drop died within 24-hr (n = 3) and 48-hr (n = 4). Only one mouse survived until day 7.	All 8 eyes exhibited: separation and/or erosion of the epithelial layer, stromal degeneration, anterior uveitis, and cataract formation.	All 8 eyes exhibited: choroiditis, moderate to severe retinal degeneration, retinal detachment, and posterior uveitis.
**2.)** Ipsilateral eye received PAO 100 µg / 5 µl ethanol + 1 % BAL 5 µL, 5 min after PAO. Contralateral eye received 5 µL of 100 % ethanol.	8*	All animals with PAO injury survived post BAL treatment. At 48-hr, ½ of the mice were euthanized, (n = 4). Then at day−7, the remaining ½ of the mice were euthanized, (n = 4).	There was a general loss of corneal epithelium in 3/6 eyes. Visible anterior chamber reaction in 3/6 eyes, Stromal dystrophy in 3/6 eyes, Cataract 1/8 eyes, and two samples had only lens sections, due to improper tissue collection.	There were normal posterior segment sections in 6/6 eyes.

* Eight mice were used in the test, 4 male and 4 female.

#### 4.3.1 PAO induced early death, and ocular injury is prevented with 1 % BAL

All the mice that received 1 % BAL 5 min after being injured with PAO survived, they were active and feeding normally, and they were euthanized at 48-hr and 7-days post injury. Although histological examination showed corneal epithelial loss and anterior chamber reaction in some mice, the posterior segment of all eyes was rescued from retinal degeneration (Table1), Additionally, OCT data at 48-hr shows that the thickness of PAO + BAL treated corneas is significantly higher than ethanol and PAO treated corneas, (Fig. 4, Fig. 5 & 6).

#### 4.3.2 Ethanol treated contralateral eye recovers from transient corneal injury

Ethanol instillation caused severe eye irritation resulting in vigorous eye wiping (Fig. 5). It was significantly higher than after PAO and PAO + BAL response. Corneas treated with ethanol were positive for fluorescein at 30 min, most eyes recovered at 48-hr, and all eyes recovered by 7-days, (data not shown).

## 5 Discussion

Lewisite (LEW), dichloro (2-chlorovinyl)-arsine, is an extremely deadly arsenical vesicant chemical warfare agent developed by Dr. W. Lee Lewis in 1917, and it was first weaponized prior to the World War II, with BAL being the only FDA approved systemic therapy for lewisite and other arsenical toxicity, despite its reported side effects [23]. The human eye is one of the most exposed organs and therefore highly susceptible to chemical toxicity, apart from the skin and lungs. The consequences of exposure can lead to irreversible blindness, and unfortunately there is no approved therapy for ocular lewisite toxicity [24]. In view of the restrictive regulatory criteria of working with Lewisite, we chose to replace Lewisite with PAO, based on previous studies, which ascertain that it is suitable as a surrogate for Lewisite [24], [25].

Considering the very rapid ocular absorption of Lewisite [8], we hypothesized that a suitable decontaminant should ideally meet the speed of the agent; the lipophilicity of BAL renders it with quick penetrability though the cornea and considering it is the sole approved therapy for systemic arsenical toxicity, we planned to move it forward to topical ocular application in this case. BAL has been known to be a very effective chelating agent for cases of extreme heavy metal contamination. Because of this, we also investigated the binding efficiency of BAL with arsenic and with the essential cellular cations, calcium, and zinc, using calcium chloride and zinc chloride, respectively. Interestingly, our results showed an effective binding of BAL with arsenic trioxide at 7.5 pH and negligible interaction with calcium and zinc. Cavanillas, S. et. Al., [26] have also reported using ITC, for testing the binding efficacy of arsenic with chelators which are analogs of BAL. Zinc and calcium play an important role in cell proliferation, differentiation, motility and defense against free radicals, and our study shows negligible binding of BAL with calcium and zinc, which implies BAL may not impede corneal healing by chelating these ions. This paves way for selecting BAL as a suitable decontaminant for arsenical chemical warfare agent exposure. Topical use of 5 % BAL on the rabbit eye has been previously reported to be effective for lewisite injury, but with notable drawbacks [8]. However, there have been no follow-up reports on improving the side effects caused by BAL. Therefore, we tested the dose dependent influence of BAL on ex-vivo mouse cornea.

We observed a significant increase in corneal thickness and fluorescein retention with 5 % BAL versus 0.1 % and 1 % BAL topical drops, (Fig. 3, Supplemental Figure S1, Panels 1–4). Previous researchers have also reported a drug layer over the rabbit cornea with an increasing concentration of BAL [8]. As a result, we selected 1 % BAL as the therapeutic concentration for our studies. In the ex vivo eyes we chose a lower dose of PAO [24] to induce the injury, considering that a longer retention on the surface in the absence of lacrimation and blinking reflex will incite similar pathogenesis. Ex vivo mice corneas were injury induced with 25 µg of PAO and treated with 1 % BAL after 5 min, slit lamp biomicroscopic examinations and OCT data showed rescue in the intensity of injury versus the untreated PAO injured corneas.

As for the in-vivo, it was designed with double the dose than a previous study [24]. There is an extensive knowledge gap regarding the dose response and toxic effects for lewisite [23]. A recent study reports dose dependent increase in the metabolic toxicity following lewisite exposure to the cornea in a rabbit model [27]. Zhylkibayev et al. (2023) applied 50 µg of PAO for 3 min by using a filter paper soaked in drug solution. In our study, we did not use such prolonged exposure with soaked filter paper application. Instead, a higher dose of 100 µg was used in a drop, which is expected to be retained less well than in prior study. Further, while drug is better retained in ex vivo eyes, it drains readily from eye surface in vivo. Therefore, compared to ex vivo studies, a higher dose was chosen for our in vivo studies. We tested the ocular toxicity with a high dose (100 µg) of PAO and evaluated mitigation of the pathogenesis with much lower concentration of BAL than previously used. We chose to treat the PAO injured eyes with 1 % BAL 5 min after injury based on insights from a previous study [8], which states that the penetrability of lewisite is rapid. Hughes et al. (1947) observed that lewisite penetrates the eye rapidly within two minutes and causes irreversible loss to the eye structures within ten minutes. Therefore, a 5 min intervention was undertaken in this study.

Curiously and highly unanticipated, seven out of the eight mice died within 48-hr of the exposure, while the animals that received 1 % BAL concomitantly with PAO, survived and remained normally active. To our knowledge, this is the first study that reports deaths due to ocular exposure of arsenical compounds in mice, whereas mortality due to dermal exposure to lewisite and its prevention with BAL has been reported earlier in rats and guinea pigs [28]. The cause of death in this study could be either due to the intense pain of the injury or systemic toxicity due to the absorption of PAO, to determine the exact cause would require further investigation.

The clinical and histopathological findings of the mice eyes at the 48-hr time point shows some attenuation of adverse response in the anterior segment and apparent rescue of the posterior segment was seen in all eyes treated with 1 % BAL. As a result, we hypothesized that neutralization of PAO by BAL on the ocular surface, prevented its penetration into the posterior segment and thereby circumventing the pathological changes incited by PAO, previous observations show, lower doses of PAO injury on the ocular surface has resulted in reduction of ERG amplitude, retinal degeneration, and blindness in mice [24]. We have observed severe retinal degeneration and detachment with the concentration of PAO used in our study, which was averted in the BAL treated eyes. Although corneal epithelial loss, corneal haze and lid edema were observed in both, BAL treated and untreated eyes, the corneal contour and thickness were retained, and anterior chamber reaction was minimized in BAL treated eyes. The cause of corneal epithelial injury in the treated animals could be due to the fast action of PAO before receiving BAL, or the drug combination of PAO + BAL may have resulted in localized epithelial erosion, moreover side effects of BAL itself cannot be ruled out, dermal sensitization with 1 % BAL resulted in transient dermatitis, which disappeared within one week [29]. Therefore, it is important to test various concentrations of BAL on the ocular surface with and without arsenic injury for acute and delayed response. One limitation of our study is that the left and right eyes received different treatments and considered independent. Another limitation is the use of qualitative histology data.

It is worth noting that the corneal epithelium possesses the capacity to regenerate [30], [31] and mainstay treatment with lubricants, anti-inflammatory, and antioxidant topical medications may improve the reparative process, and this should be investigated with the current injury and therapy protocol. In this study, apart from topical antibiotics, no other supportive medication was administered. Prevention of death and preservation of the retinal architecture were profoundly exciting observations of the study with 1 % BAL as topical therapy since retinal regeneration and repair are extremely challenging. It is crucial to highlight that BAL is the approved therapy for systemic lewisite toxicity. The drawbacks associated with systemic therapy of this potent chelator are not pertinent for topical use in the eye. Importantly, the efficacy of BAL in mitigating lewisite induced surface toxicity scores is superior to its analogs [3]. Therefore, further investigations are warranted to steer the drug towards the possibility of safer clinical application in the eye.

## 6 Conclusions

Based on this pilot study and reports from other researchers, BAL is a choice for anti-lewisite surface decontamination of the eye. In future studies, the drug may be improved further through formulation and combination therapy approaches to establish a clinically relevant therapy for lewisite and other arsenical vesicants to decontaminate the eye after exposure.

## CRediT authorship contribution statement

**Sarbani Hazra:** Writing – review & editing, Writing – original draft, Visualization, Methodology, Investigation, Formal analysis, Data curation, Conceptualization. **Uday B. Kompella:** Writing – review & editing, Supervision, Resources, Project administration, Funding acquisition, Conceptualization. **Aditya Konar:** Writing – review & editing, Visualization, Methodology, Investigation, Formal analysis, Data curation, Conceptualization. **Robb Welty:** Writing – review & editing, Visualization, Methodology, Investigation, Formal analysis, Data curation.

## Declaration of Generative AI and AI-assisted technologies in the writing process

Generative AI and AI-assisted technologies were not used in the preparation or revisions of this manuscript.

## Funding

This research received no external funding, but the work was supported by internal funds of Dr. Uday Kompella at the University of Colorado Skaggs School of Pharmaceutical Sciences, Anschutz Medical Campus, Aurora, CO 80045, USA.

## Declaration of Competing Interest

The authors declare that they have no known competing financial interests or personal relationships that could have appeared to influence the work reported in this paper.

## Data Availability

All data is supplied within the article
