# Supplementary material for: British anti-Lewisite (BAL) reduces the severity of systemic and local responses of the eye after exposure to the chemical warfare agent surrogate for Lewisite, phenylarsine oxide (PAO)
Source: Toxicol Rep. 2025 Oct 28;15:102153. doi: 10.1016/j.toxrep.2025.102153 (PMC12648705; doi:10.1016/j.toxrep.2025.102153)
Supplement: Supplementary file 1 — Supplementary material [file mmc1.docx]

**Supplementary Material**


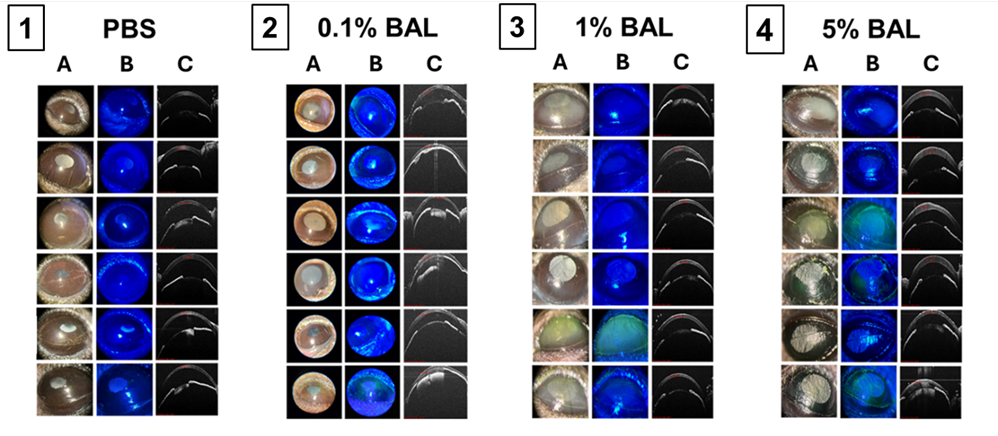


**Supplemental Figure S1, Panels 1 - 4.** **Corneal Slit lamp biomicroscope and OCT images of six ex vivo mice eyes treated with PBS (1), 0.1% BAL (2), 1% BAL (3), and 5% BAL (4).** Images are arranged in three columns; (A) and (B) show slit lamp biomicroscopy using bright light and cobalt blue settings, and (C) shows the OCT images. 1.) Eyes treated with PBS showing normal corneal clarity, absence of epithelial loss, and normal corneal thickness in many of the mice eyes. In 1 out of six eyes (row 6) the cornea exhibits punctate fluorescein retention and maintenance of corneal clarity, while the OCT images show the normal corneal architecture, 2.) Eyes treated with 0.1% BAL showing mild corneal haziness, lack of fluorescein retention, but normal corneal thickness in many of the eyes. In 1 out of six (row 6) show fluorescein retention in the cornea with mild corneal haziness which is apparent in all mouse eyes while OCT images still depict normal corneal architecture, 3.) Eyes treated with 1% BAL showing obvious corneal haziness, lack of fluorescein retention, but normal corneal thickness in most of the mice eyes. Many of the eyes showed no retention of the fluorescein except for rows five & six. A drug sheet formation and corneal haziness is evident in all eyes. The OCT images once again show the normal corneal architecture, 4.) Eyes treated with 5% BAL showing conspicuous corneal haziness, fluorescein retention and enhanced corneal thickness in some of the eyes. Slit lamp images show diminished corneal clarity due to the drug sheet formation on the corneal surface. The eyes are positive for retention of fluorescein dye in rows 3, 4, and 6. In the OCT images there is a visible increase in corneal thickness.

**Supplemental Figure S2, Panels 1 - 2.** **Slit lamp, OCT, and histological images of four ex vivo whole or cross-sections of mice eyes injured with PAO, and PAO / BAL.** Images are arranged in five columns; Columns (A) and (B) are images from a slit lamp bio-microscope obtained under bright light and cobalt blue settings, Column (C) shows OCT images, Columns (D) and (E) show whole eye and cross-section histology images. 1.) Eyes treated with 25 µg PAO. Slit Lamp biomicroscope images show positive fluorescein retention in 4 of 4 corneas treated with PAO, while OCT images show a reduction of the corneal thickness. Histopathological sections show loss of corneal epithelium in tune with regions of fluorescein retention in the clinical images. These sections show regions of corneal epithelial loss in 4 of 4 treated eyes. 2.) Eyes treated with 25 µg PAO and 1% BAL showing reduced loss of corneal epithelium, reduced fluorescein retention, and preserved corneal thickness. The concomitant use of PAO and BAL shows apparent lack of fluorescein dye in 1/4 corneas (row 4). Very mild dye retention was noticed below the drug sheet formation in one eye (rows 1 and 3), and apparent dye retention and peeling of epithelium in the fourth cornea (row 2). The OCT images depict preserved corneal thickness. The regions of the loss for corneal epithelium in histological sections corroborate the clinical observations.
